# Sigma-1 receptor agonist fluvoxamine for postoperative delirium in older adults: report of three cases

**DOI:** 10.1186/1744-859X-9-28

**Published:** 2010-06-24

**Authors:** Tsutomu Furuse, Kenji Hashimoto

**Affiliations:** 1 Department of Psychiatry, Asahikawa Red Cross Hospital, Asahikawa, Japan; 2 Division of Clinical Neuroscience, Chiba University Center for Forensic Mental Health, Chiba, Japan

## Abstract

**Background:**

Postoperative delirium is a topic of great importance in the geriatric surgical specialty. Although antipsychotic drugs are the medications most frequently used to treat this syndrome, these drugs are associated with a variety of adverse events, including sedation, extrapyramidal side effects, and cardiac arrhythmias. Drug treatment for postoperative delirium requires careful consideration of the balance between the effective management of symptoms and potential adverse effects.

**Methods:**

We report on a Japanese woman (an 86-year-old (open reduction and internal fixation of the right femoral neck fracture), and two Japanese men (an 86-year-old (abdominal aortic aneurysm stent grafting), and a 77-year-old (right upper lobectomy due to lung tumour)) in which the selective serotonin reuptake inhibitor and sigma-1 receptor agonist fluvoxamine was effective in ameliorating the postoperative delirium of these patients.

**Results:**

Delirium Rating Scale scores in these patients dramatically decreased after treatment with fluvoxamine.

**Conclusions:**

Doctors should consider fluvoxamine as an alternative approach to treating postoperative delirium in older patients in order to avoid the risk of side effects and increased mortality by antipsychotic drugs.

## Background

Postoperative delirium is a common and deleterious complication in older patients following a major operation. The recognition and treatment of postoperative delirium is critically important because postoperative delirium is associated with increased morbidity and mortality, prolonged hospital stays, and cognitive deterioration [1-3]. Antipsychotic drugs have been widely used for the treatment of delirium. However, antipsychotic drugs are associated with a variety of adverse events including sedation, extrapyramidal side effects, and cardiac arrhythmias. In addition, there is an elevated risk of mortality in older patients treated with atypical antipsychotics [4,5]. Although the pathophysiology of delirium is not fully understood, current evidence suggests that drug toxicity, inflammation and acute stress responses can all contribute to disruption of neurotransmission, and, ultimately, to the development of delirium [6].

The endoplasmic reticulum protein sigma-1 receptors play a key role in Ca^2+ ^signalling and cell survival, and have been shown to regulate a number of neurotransmitter systems in the brain [7-12]. The selective serotonin reuptake inhibitor fluvoxamine is a very potent agonist at sigma-1 receptors that are implicated in the pathophysiology of neuropsychiatric diseases as well as cognition [10-12]. Recently, we reported the cases showing that fluvoxamine was effective in the treatment of delirium in the patients with Alzheimer disease [13] and intensive care units [14]. We proposed a hypothesis that fluvoxamine may be effective in the treatment of delirium. Here, we report three cases in older adults where fluvoxamine was effective in patients with postoperative delirium.

## Methods

### Consent

The three patients deteriorated mental status made the informed consent procedure reasonably difficult. To this extent consents were obtained from the patient's next of kin and efforts have been made so that patient's identity remains anonymous and there is no reason to think that the patients or their family would object to publication.

## Results

### Case reports

#### Case 1

An 86-year-old Japanese woman was admitted to a hospital emergency room after falling at a health and mental centre for older patients. The patient was diagnosed with a right femoral neck fracture on X-ray examination, and she received emergency open reduction and fixation. She became overly excited while waking from anaesthesia, and was referred to the hospital's department of psychiatry. She was disoriented and agitated. To treat her postoperative delirium, she was administered fluvoxamine (50 mg, twice a day) and flunitrazepam (1 mg, at night). The next day she did not remember that she had received an operation, and she was unhappy with the cast on her leg. Therefore, the fluvoxamine was increased to 100 mg (twice a day), since there were no gastrointestinal side effects. At 2 days after the first treatment, fluvoxamine was increased to 150 mg (twice a day). Her sleep disturbance improved, and her Delirium Rating Scale (DRS) [15] score decreased dramatically, from 27/32 to 13/32. After recovery, her Mini-Mental State Examination (MMSE) [16] score was 8/32.

#### Case 2

An 86-year-old Japanese man had been admitted to a health and mental centre for older patients for 2 years. The patient was diagnosed with abdominal aortic aneurysm, and was a candidate for surgery. He underwent the operation using a custom-made stent graft for abdominal aortic aneurysm. After his operation, he had insomnia and was in a seditious state. Therefore, he was referred to the hospital's department of psychiatry. He was disoriented and agitated, and was diagnosed with postoperative delirium. To treat this, he was administered fluvoxamine (50 mg, twice a day) and lorazepam (0.5 mg, at night). The day after the first treatment, his sleep disturbance improved, and his DRS score decreased dramatically, from 19/32 to 8/32. After recovery, his MMSE score was 10/30.

#### Case 3

A 77-year-old Japanese man was diagnosed with right upper lobectomy due to a right lung apex tumour, and underwent surgery to remove the tumour. His cardiorespiratory dynamic state was stable, but he became excited after the tracheal extubation. Therefore, he was referred to the hospital's department of psychiatry. He was disoriented and agitated, and was diagnosed with postoperative delirium. To treat his postoperative delirium, he was administered fluvoxamine (50 mg, twice a day) and flunitrazepam (1 mg, at night). The next day fluvoxamine was increased to 100 mg (twice a day), since there were no gastrointestinal side effects. At 2 days after the first treatment, his DRS score decreased dramatically, from 20/32 to 10/32. After recovery, his MMSE score was 9/30.

## Discussion

To our knowledge, this case report is the first to demonstrate that fluvoxamine is rapidly effective for treating postoperative delirium in older patients. Nonetheless, a randomised double-blind, placebo-controlled study of fluvoxamine will be needed to confirm its efficacy for the treatment of postoperative delirium in patients. Recent findings suggest that sigma-1 receptors might be involved in the different mechanisms of some selective serotonin reuptake inhibitors (SSRIs), and that fluvoxamine is a potent sigma-1 receptor agonist [10-12]. Currently, it is unclear whether sigma-1 receptors were involved in the mechanism underlying the beneficial effects of fluvoxamine against the postoperative delirium of these patients. In order to confirm the role of sigma-1 receptors in the treatment of postoperative delirium, a randomised double-blind, placebo-controlled study of selective sigma-1 receptor agonists (for example, cutamesine (SA4503)) in patients with postoperative delirium would also be of interest.

In all the patients reported on here, low doses of flunitrazepam or lorazepam were used for the treatment of insomnia since these drugs are considered to be the most effective benzodiazepine hypnotics. Therefore, we cannot exclude a possible contribution of these hypnotics on the efficacy of fluvoxamine for delirium. A further study of fluvoxamine alone will be necessary.

Delirium is theorised to be a neurobehavioural manifestation of imbalances in the synthesis, release, and inactivation of a number of neurotransmitters that normally control cognitive function, behaviour, and mood [6]. Given the role of sigma-1 receptors in the regulation of a number of neurotransmitters as well as in cognition [7-12], it is likely that sigma-1 receptor agonist may be involved in the fluvoxamine's mechanisms of action, although a further study will be necessary.

Drug treatment for postoperative delirium requires careful consideration of the balance between the effective management of symptoms and potential adverse effects. As mentioned above, there is an elevated risk of mortality in older patients treated with atypical antipsychotics [4,5], suggesting that the widespread use of atypical antipsychotic drugs in older adults should be re-evaluated. Therefore, the sigma-1 receptor agonist fluvoxamine may serve as an alternative treatment option for older adults with postoperative delirium, although further detailed studies on the role of sigma-1 receptors in postoperative delirium are necessary.

## Conclusions

This case report suggests that fluvoxamine could be an alternative approach to treating postoperative delirium in older adults because of the risk of extrapyramidal side effects and increased mortality by antipsychotic drugs.

## Competing interests

The authors declare that they have no competing interests.

## Authors' contributions

TF contributed to the clinical and rating evaluations during the follow-up periods. KH conceived of the study and participated in its study and coordination. Both authors read and approved the final manuscript.
